# The feminization of the medical work force, implications for Scottish primary care: a survey of Scottish general practitioners

**DOI:** 10.1186/1472-6963-6-56

**Published:** 2006-05-10

**Authors:** Brian McKinstry, Iain Colthart, Katy Elliott, Colin Hunter

**Affiliations:** 1 University of Edinburgh, Community Health Sciences: GP Section, 20, W Richmond Street, Edinburgh EH8 9DX, UK; 2 NHS Education for Scotland, The Lister, 11, Hill Square, Edinburgh EH8 9DR, UK; 3 Skene Medical Centre, Westhill Dr, Westhill, Aberdeenshire AB32 6RL, UK

## Abstract

**Background:**

The number of women working in general practice internationally has been steadily rising. In Scotland there have been concerns that such a change may lead to increased part-time working and subsequently to a fall in available general practice manpower despite an apparently rising overall number of general practitioners. However, there is very little information on the actual hours worked by men and women general practitioners or on the types of work they are undertaking.

**Methods:**

Anonymous workload questionnaires of all Scottish general practitioner principals and non-principals

**Results:**

Response rates for general practice principals and non-principals were 67.2% and 65.2% respectively. Male principals spent on average 18% more time on general medical services (GMS) and 50% more time on non-GMS activities (such as teaching, specialist sessions, administration and research) than women (both p <0.01). This difference was similar for non-principals. In no age group did the hours worked by women doctors approach that of male doctors.

**Conclusion:**

Women doctors in primary care in Scotland work fewer hours in all age groups than their male counterparts. The rapidly increasing proportion of women in general practice may lead to an increasing shortfall of medical availability in the future if current work patterns are maintained. Further longitudinal research is required to establish this and man-power planning is required now to address this. More worryingly auxiliary activities such as teaching and administrative duties are not being taken up by women. This may have serious implications for the future development of the specialty in Scotland.

## Background

Faced with an aging population and the falling popularity of general practice as a career there have been concerns that the future Scottish general practitioner (GP) work force may be insufficient to manage a rising clinical workload [1]. As in other countries [2], there has been a marked increase in the proportion of general practitioners who are women, partly due to a falling interest in general practice by men and partly to the steadily increasing proportion of women medical graduates [3]. This has led to further concerns that the capacity of the workforce might be reduced by part-time working in Scotland. While raw numbers of principals and non-principals in Scotland had been rising [4], little is known about how much time each of these groups is spending on general medical services (GMS) or other activities nor what their future career intentions are.

General practice in the UK is largely managed in partnerships of GPs with a few working single-handedly [5]. Up until April 2004 most of these general practitioners contracted to provide general medical services for local primary care organisations and were known as principals. Their remuneration was based on the size of the patient list they looked after and on payments for a small number of item of service fees. As independent contractors they could take other employment opportunities for example as GP educators, in primary care administration and private health care. Individual remuneration within partnerships was agreed on the basis of day-time and out-of-hours workload. Some of these doctors had entered into a special type of contract with primary care organisations to provide Personal Medical Services under which they contracted to achieve specific targets in addition to GMS.

Another group of doctors known as non-principals did not usually have a direct contract with a primary care organisation but worked as locums, assistants or on the doctors retainer scheme (a scheme to maintain doctors with domestic commitments in practice) and were employed usually on a sessional basis by GP principals (a session is a half-day roughly 3.5–4 hours). These doctors generally spend less time on the managerial and strategic planning aspects of general practice and they were largely made up of relatively young women. They too were free to take up other types of employment. In April 2004 these titles were re-designated with all doctors providing primary care services now called performers and those with contracts to provide services with the local primary care organisations additionally called providers. However despite the change in name their day-time roles remained largely the same and work patterns were maintained. Out-of-hours responsibilities passed away from GP principals to primary care organisations in April 2004.

Current information on workload relies largely on contractual information which formerly divided principals into those working in to part-time (two categories), job share or full time which was defined as more than 26 hours per week. Information on non-principals was largely restricted to those who worked on the retainer scheme and a small group of practice assistants. Little at all is known about the workload contribution of the increasingly large number of doctors who work as locums in primary care.

While it was largely assumed that doctors contracted to work more than 26 hours were working considerably more than this, it is not known to what extent doctors were working either above or below their contracted hours or if some of the contracted hours were being taken up either with National Health Service (NHS) related activity (for example working for local health care co-operatives), education, research or private medical work. The last re-organisation of the health service (1991) saw many doctors take roles in NHS management and in specialist clinics and there has been a shift of medical undergraduate education to the community.

Although the number of full-time equivalent GP principals in Scotland has been steadily rising, this increase is largely due to women doctors [6]. It has not been clear if "full-time" women doctors contribute the same number of hours as "full-time" men nor what their work pattern was in relation to non-GMS but NHS related work.

Information on workload is very sensitive as it may reveal a situation where some doctors who may be paid as 'full-time' were in reality spending only half of this time on NHS related work. Potentially, previous non-anonymous surveys may have resulted in a perception of workload that was inflated because of concerns on the part of some respondents about revealing contractual non-adherence[7].

In addition there is very little information on future work intentions. While there have been studies on retirement intentions [8-10], these have not always included the non-principal groups and there has been relatively little work on how doctors anticipate their workload changing in the future. This is particularly important for the large number of doctors currently working part-time, on retainer schemes or as locums. Likewise it is important to determine if there are differences between men in women in terms of their long term commitment to service provision in relation to their retirement plans.

The aim of this study was to discover how male and female general practice performers (formerly general practice unrestricted principals (henceforth referred to as GP principals) and non-principals) divided their time between general medical service (GMS) activity and other activities such as teaching and administrative tasks. In order to obtain an honest picture of current GMS and non-GMS workload we decided to use an anonymous survey method. As out-of hours work was shortly to be contracted out we decided to restrict our survey to in-hours work.

## Methods

### Designing the questionnaire

We realised that busy professionals would be unlikely to be able to devote a large amount of time to completing a questionnaire on workload. We aimed therefore for a questionnaire which took no more than five minutes to complete and which covered only two sides of A4.

The main components of the principal questionnaire (see Additional file 1) were: information on recent contract status; partnership share of profits (as this was considered to be an alternative description of workload share in relation to full-time); a description of 'in-hours' current workload broken down into GMS and non-GMS work; perception of change in workload in the last five years; expectation of change in workload and number of session in the next 2 years; retirement intentions and demographic data.

The non-principal questionnaire (see Additional file 2) also contained questions on GMS and non-GMS workload, the titles under which they worked (e.g. locum, assistant, retainer etc.), their career intentions over the next 5 years (e.g. locum work, becoming a principal) including to intention to stay in the NHS and in the UK and demographic data.

During construction both questionnaires were piloted on 4 occasions until we were satisfied that the questionnaires were both easy to understand and complete.

### Identifying GP performers

All general practice principals in Scotland were identified from databases held by Information and Statistics Division of NHS Scotland (ISD) and non principals from accrued Supplementary Medical Lists derived from Scottish Primary Care Trusts. Because of the way it was assembled, and the highly mobile nature of the doctors on it, this non-principal database was known to be imperfect. It contained doctors who while trained in general practice no longer practiced (for example regional directors), some doctors who had registered for work in more than one trust appeared twice, some had gone abroad and some had become principals since the list had been created. In both databases some doctors had retired or were on long term sick leave.

### Distributing the questionnaires

In May-June 2004 all GP principals and non-principals in Scotland from the 'cleaned' databases were sent the questionnaire along with a covering letter which outlined the purpose of the study and reassured the participants of complete confidentiality. Enclosed with the questionnaire was a reply paid postcard which participants were asked to return by separate post to inform us that they had completed the survey. Four weeks later those who had not returned a card were sent a reminder.

### Analysis

Data was entered into SPSS [11]. Comparisons were made using chi-square and Mann-Whitney tests where appropriate.

## Results

### Response rate and data quality

The response rate for the GP principal questionnaire was 2541/3783 (67.2%) and for the non-principal questionnaire 749/1149 (65.2%). While the GP principal response rate is reasonably accurate great caution must be used in interpreting the non-principal response rate. This group of doctors is highly mobile with many retiring from practice, leaving the country or giving up general practice to work in other parts of the NHS (over 200 indicated they had plans to behave similarly in the next 5 years in this sample group). It is very likely that the non-principal response rate is a higher proportion of those non-principals currently working in general practice in Scotland than the figures here reported suggest. We were able to compare the demography of most of our respondent samples with figures from ISD. Age and sex data were not available for the whole of the non-principal sample at the time of the study from ISD, but sex data was available for those doctors who have contracts (i.e. associates/assistants and GP retainees). Where they could be compared the demographic pattern was almost identical for the respondents and the whole Scottish database.

#### Contractual status of GP principals at the end of the previous GMS contract and current workload

**Figure 1:**
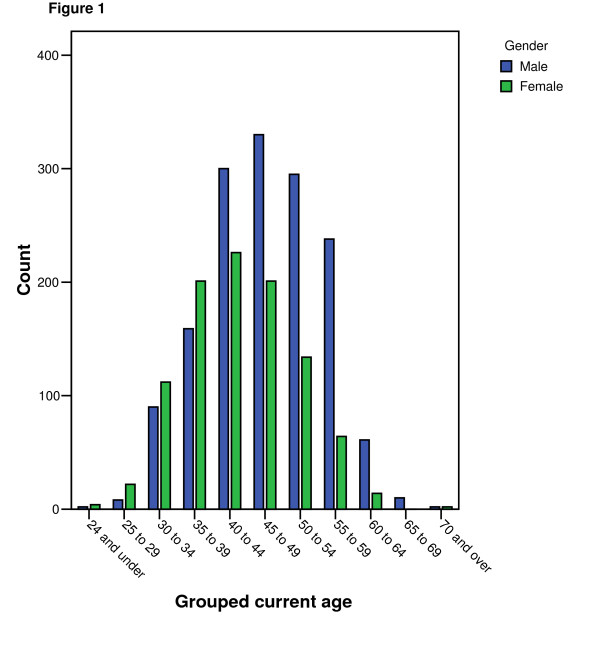
shows the age sex distribution of Scottish general practice principals.

Figure 1 shows the age sex distribution of Scottish general practice principals. In general women outnumber men in the age groups below 40 years. Men are largely concentrated in the 45 and over age groups. Table 1 shows that more men than women were contracted to work more than 26 hours a week (p < 0.01)

However, although 66.7% women are described as being 'full-time' in terms of the old GMS contract, in fact only 37.3% were paid a full share of partnership profits compared with 75.7% of males. "Full-time" males (>26 hours) provided 8.1 sessions of GMS while their female equivalents provided 7.5 sessions (p < 0.001). Table 2 shows the actual reported number of sessions worked by men and women with considerably fewer women working more than 8 sessions.

Table 3 shows how the number of sessions carried out by men and women varies with age. The average number of sessions worked by women does not equal that of males in any age group.

Table 4 shows how this time is made up. Men spend 7.87 sessions on average on clinical GMS and 1.1 sessions on other NHS related non-GMS activities while women spend 6.68 sessions on GMS and 0.73 sessions a week on other NHS activities. Women contribute to all aspects of general practice work, but in general concentrate a little more on NHS clinical GMS sessions than do men. All these differences are significant at <0.01 except for teaching medical students (<0.05) and medical research (NS). Doctors above and below the age of 40 do not work significantly different hours.

#### Future plans and perceptions of future workload

Of those GP principals who were not planning to retire in the following 2 years, 73 (3.1%) planned to increase their sessions by an average of 1.5 sessions per week and 288 (13.3%) planned to decrease their sessions by an average of 2.0 sessions a week. Women were more likely than men to be planning an increase in sessions (p < 0.05). 178 (7%) planned to retire in the next two years. Doctors of both sexes planned to retire on or before 60. The mean planned age of retirement for women was 58.8 and for men 59.9.

## Non-principal survey

Responses from non-principals reflected the type of post they held. When appropriate, data is presented grouped then broken down by the larger groups of non-principal type.

The age-sex distribution of the respondents is shown in table 5. In keeping with the principal data this shows the increasing contribution of women to the workforce.

### Numbers and types of non-principal

Table 6 shows the type of non-principals that responded to the survey. The majority of the respondents worked as locums and retainees with smaller numbers of associates and assistants. Table 5 shows the age sex distribution of the respondents

### Contribution to in-hours clinical general practice

Respondents contributed an average of 5.15 (SD2.49) sessions per week to clinical general practice. Exclusive GP retainers contributed on average 3.5 (SD 0.8) and exclusive locums 5.7 (SD 2.6) sessions per week. Men provided on average 5.8 (SD 2.8) sessions and women 4.9 (SD 2.3) sessions per week. Doctors over the age of 35 infrequently worked more than 5 sessions.

### Contribution to non-general practice NHS work

36 (14.8%) men and 66 (13.3%) women undertook other NHS work outside of general practice mainly hospital sessions and teaching. Those that took part in other NHS work spent on average 2.8 sessions per week on it.

### Non NHS work

More men (66 (28.6%)) than women (72 (15.1%)) undertook non-NHS sessions (p < 0.001). These were very wide ranging, the commonest being occupational medicine (32), DSS medical work (26), prison medical work (4) and sports medicine (4). Those that took part in non-NHS work spent on average 10.5 sessions per month on it.

### Career intentions

Respondents were asked what their likely career intentions were in the next five years. There were significant differences in career intention between men and women, while the majority of both sexes had decided against a traditional partnership career path a greater proportion of women were intending to become partners (196 (39.6%) v 72 (29.5% p <0.01). Relatively more men opted to remain as locums or leave the NHS. However decisions to leave GP, NHS and Medicine were heavily weighted by those intending to retire (22 respondents) who were mainly male. Twelve respondents gave reasons for leaving general practice related to the stress of the job, the remainder intending career changes.

### Likely change in clinical commitment to general practice

Respondents were asked if they thought their commitment to general practice was likely to change in the next 5 years. Most thought it would remain the same, however, women were more likely than men to think it would increase (125/439 (28.5%) v 38/198 (19.2%), p < 0.01).

Clinical commitment to non-GP NHS and non-NHS roles was considered likely to remain stable overall.

## Discussion

Clearly these figures rely exclusively on self report of work-load. Doctors like most people are unlikely to minimise their workload, but unlike other surveys which have been carried out specifically with calculation of remuneration in mind this was an anonymous survey which sought to inform the need for future GP training. Previous work in this area in Scotland has been dogged with a poor response [12]. Response rates in this study were good for a postal survey, but care has to be taken when extrapolating the results to the whole population. Although the age-sex distribution of respondents is gratifyingly close to data we know to be accurate, this need not imply that the workloads and future plans of non-responders will be the same as those of responders. Additionally the data provides only a cross-sectional snapshot of Scottish GPs. It is possible that current patterns of working may not persist and for example women now currently in their 30s will choose to work longer hours in their 40s and 50s than women of that age currently do. Indeed women working part-time in this survey were more likely than men to say they planned to increase their hours. Longitudinal studies are required to explore such phenomena.

Our data seem to suggest that the previous assumption that full-time doctors worked nine GMS sessions is an over-estimate and this is particularly true for women doctors. Overall doctors provide 7.4 clinical GMS sessions and an additional 0.9 session of NHS, educational or research related work. In comparison with previous surveys the perception of time actually spent on GMS appears to have recently increased [7].

Over recent years the rise in non-principals (largely female) has been considered to be boosting the work force, however, their average GMS commitment is 5.15 sessions per week. This data includes accurate data on the contribution of GP locums for the first time. Perhaps of some concern is the fact that most of these largely female doctors do not see themselves following the partnership route in general practice, but intend to stay on as locums or assistants of some kind. This clearly will have implications for the way general practice is managed with the current structure of equal partnerships giving way to the models seen in the legal and accountancy sectors of a small number of executive partners aided by salaried assistants. It appears that this latter group are likely to be dominated by female doctors. This has also been found in other European countries [2,13].

Additionally and perhaps more worryingly, women are contributing only about 60% of the activity of men in aspects of general practice involved with its development such as training, teaching and research. It is not clear why women are not more involved in non-GMS activities. This may be down to a prioritisation between home and work life, active discrimination or unintentional "glass-ceiling" effects. It is a phenomenon described by other researchers [12] and poses a considerable challenge in maintaining Scotland as a first rate centre for general practice research and development.

Given that most of the current GPs in Scotland intending to retire within the next 10 years (mainly male and full-time) are likely to be replaced by a mainly female and less than full time work force there are concerns about the numbers of doctors currently being trained and retained in general practice in Scotland (personal communication David Blaney, 2005). With the likely medical shortfall expected to start to become apparent about five years from now, hard choices on what constitutes medical primary care may have to be made. It is likely that the generic family practitioner of today is going to become a luxury. Such concerns are also being voiced internationally where women are becoming an increasing part of the general practice workforce [2,13-15].

It is not clear why women opt for part-time working. There is evidence that women are more likely to change their preferred career to general practice than men [16]. A desire to combine a career with family life is the standard reason and general practice is a specialty which provides sufficient flexibility to do this. However, it is not clear how much of this decision is by choice and how much is down to a subtle coercion by societal or male partner expectation leading to concerns that women or not fulfilling their potential [17]. Additionally, there is a growing perception, somewhat supported by our figures of an increasing trend for males also to work part-time and to be reluctant to commit to a life-long career [18].

The Kerr report[19] has emphasised the pivotal role primary care has now and will have in the future of the Scottish NHS. In order to maintain even the current level and type of service clearly more doctors will need to be trained, retained in Scotland or encouraged to come from other countries. Alternatively, as is already happening, nurses and other paramedical staff may have to increasingly perform tasks once carried out by doctors. Out-of-hours models of triage may be adapted for in-hours work with medical staff seeing the more complex patients, acting increasingly and necessarily in semi-specialist roles. However there is little evidence on the cost-effectiveness of such substitutions and further research is required before embarking on this type of policy [20].

## Conclusion

Women doctors in primary care in Scotland work fewer hours in all age groups than their male counterparts. The rapidly increasing proportion of women in general practice may lead to an increasing shortfall of medical availability in the future if current work patterns are maintained. Further longitudinal research is required to establish this and man-power planning is required now to address this. More worryingly auxiliary activities such as teaching and administrative duties are not being taken up by women. This may have serious implications for the future development of the specialty in Scotland.

## Abbreviations

GP General Practitioner

GMS General Medical Services

NHS National Health Service

## Competing interests

All the authors are currently or have been employed by NHS Education for Scotland, the organisation responsible for GP training in Scotland.

## Authors' contributions

BM conceived and planned the research, helped analyse the data and wrote the paper. IC, entered and analysed the data and helped write the paper, KE distributed and helped design the questionnaires, entered the data, and proof-read the paper. Colin Hunter helped with recruitment and helped to plan the research and design the questionnaires. All authors read and approved the final manuscript.

## Pre-publication history

The pre-publication history for this paper can be accessed here:



## Supplementary Material

Additional File 1 Questionnaire for principals Click here for file

Additional File 2 Questionnaire for non-principals Click here for file

## Figures and Tables

**Table 1:** Contracted commitment per week of general practice principals

Contracted commitment per week	Gender		Total
	Male (%)	Female (%)	

13 to 18 hours	23 (1.6)	52 (5.3)	75 (3.1)
19 to 26 hours	86 (5.8)	234 (24.0)	320 (13.1)
More than 26 hours	1323 (89.9)	651 (66.7)	1974 (80.7)
job share*	39 (2.7)	39 (4.0)	78 (3.2)
Total	1471(100)	976 (100)	2447 (100)

Missing data 94

* Two doctors who chose to share the hours and responsibilities of a full-time partnership post in a flexible way. (As part-time posts have become more readily available in recent years this type of post has become less common)

**Table 2:** Total number of general practice principal sessions performed per week by doctors' sex

Grouped total number of sessions per week		Male	Female	Total
1 to 2	Count	2	1	3
	% within Gender	.1%	.1%	.1%
2.1 to 5	Count	71	256	327
	% within Gender	4.8%	25.8%	13.2%
5.1 to 8	Count	271	392	663
	% within Gender	18.3%	39.5%	26.8%
8.1 to 11	Count	1121	340	1461
	% within Gender	75.6%	34.2%	59.0%
11.1 to 13	Count	14	4	18
	% within Gender	.9%	.4%	.7%
More than 13	Count	4	0	4
	% within Gender	.3%	.0%	.2%
Total	Count	1483	993	2476
	% within Gender	100.0%	100.0%	100.0%

Missing data 66

**Table 3:** Average number of sessions carried out by GP principals by age and sex

	Male			Female			
Age yrs.	n	sessions	SD	n	sessions	SD	p

≤29	10	9.1	0.2	26	8.3	1.3	NS
30–34	88	8.7	1.2	110	7.2	1.9	<0.001
35–39	156	8.5	1.3	197	6.9	2.0	<0.001
40–44	291	8.8	1.3	222	8.0	1.9	<0.001
45–49	328	8.9	1.4	197	7.1	1.8	<0.001
50–54	290	8.8	1.5	130	8.4	1.7	<0.001
55–59	235	8.7	1.5	64	7.6	1.8	<0.001
60–64	60	8.4	2.0	14	7.8	2.4	NS
≥65	12	8.3	0.7	2	6.5	3.5	NS
Total	1470	8.8	1.4	962	7.2	1.9	<0.001

Missing data 44

**Table 4:** Sessions spent by general practice principals on GMS and non-GMS workload by sex

	Male			female		
	N	Mean	Std. Deviation	N	Mean	Std. Deviation

NHS clinical GMS work sessions	1491	7.87	1.69	1004	6.68	1.96
NHS clinical non-GMS work sessions	1508	0.35	0.87	1010	0.24	0.68
GP Registrar training sessions	1508	0.21	0.60	1010	0.13	0.48
Teaching medical students sessions	1508	0.12	0.41	1010	0.1	0.32
Medical research sessions	1508	0.04	0.38	1010	0.03	0.29
LHCC, PCT, admin or appraiser sessions	1508	0.27	0.78	1010	0.17	0.62
Other NHS/Univ Non-GMS work sessions	1508	0.11	0.49	1010	0.06	0.31
Non-NHS work sessions	1504	0.34	0.78	1009	0.22	0.66

Missing 28

**Table 5:** Age-sex distribution of the general practice non-principal respondents

Age		Male	Female	Total
25 to 34	Count	58	187	245
	% within Gender	24.9%	39.9%	34.9%
35 to 44	Count	54	174	228
	% within Gender	23.2%	37.1%	32.5%
45 to 54	Count	33	78	111
	% within Gender	14.2%	16.6%	15.8%
55 to 64	Count	61	26	87
	% within Gender	26.2%	5.5%	12.4%
65 to 74	Count	27	4	31
	% within Gender	11.6%	.9%	4.4%
Total	Count	233	469	702
	% within Gender	100.0%	100.0%	100.0%

Missing data 47

**Table 6:** Types of non-principal (respondents could declare more than one activity)

	male	%	Female	%	total
Locum GP	169	69.3	251	51.0	420
GP assistant	27	11.1	64	12.9	91
GP associate	22	9.0	26	5.3	48
GP fellow	10	4.1	15	3.0	25
GP retainee	3	1.2	152	30.7	155
GP other	20	8.2	40	8.0	60

## References

[B1] (2004). Building and supporting the NHS workforce. British Medical Association.

[B2] Boerma W, van den Brink-Muinen A (2000). Gender-related differences in the organizatio and provision of services among general practitioners in Europe: a signal to health care planners. Medical Care.

[B3] Lambert T, Goldacre M, Edwards C, Parkhouse J (1996). Career preferences of doctors who qualified in the United Kingdom in 1993. BMJ.

[B4] (2005). General Practice workforce 2004.

[B5] (2005). General Practice in the UK.

[B6] (2005). GMP analysis by Gender (UPEs).

[B7] (1998). Review body on doctors' and dentists' remuneration, 27th report 1998.

[B8] (2001). National Survey of GP Opinion Scotland ResultsTop-Line Report. British Medical Association.

[B9] Chambers M, Colthart I, McKinstry B (2004). Scottish general practitioners' willingness to take part in a post-retirement retention scheme: questionnaire survey. BMJ.

[B10] Sibbald B, Bojke C, Gravelle H (2003). National survey of job satisfaction and retirement intentions among general practitioners in England. BMJ.

[B11] SPSS Inc (2003). Statistical Package for Social Sciences. [11].

[B12] French F, Needham G, Walker K (2005). Towards a flexible workforce – a basis for change.

[B13] Mayorova T, Stevens F, Scherpbier A, der Velden L, van der Zee J (2005). Gender related differences in general practice preferences: longitudinal evidence from the Netherlands 1982–2001. Health Policy.

[B14] Clearahan L (1999). Feminisation of the medical workforce. Is it just a gender issue?. Aust Fam Physician.

[B15] (2002). OMA Human Resources Committee (OHRC). Position paper on physician workforce policy and planning. Ontario Ontario Medical Association.

[B16] Goldacre M, Lambert T (2000). Stability and change in career choices of junior doctors: postal questionnaire surveys of the United Kingdom qualifiers of 1993. Medical Education.

[B17] Allen I (2006). Women doctors and their careers what now?. BMJ.

[B18] Vaughan C (1995). Career choices for generation X. BMJ.

[B19] Kerr D (2005). Building a health service fit for future. Scottish Executive.

[B20] Sibbald B, Shen J, McBride A (2004). Changing the skill-mix of the health care workforce. Journ Health Serv Res Policy.

