# Supplementary material for: The feminization of the medical work force, implications for Scottish primary care: a survey of Scottish general practitioners
Source: BMC Health Serv Res. 2006 May 10;6:56. doi: 10.1186/1472-6963-6-56 (PMC1475570; doi:10.1186/1472-6963-6-56)
Supplement: Additional File 1 — Questionnaire for principals [file 1472-6963-6-56-S1.doc]

1. Under what contractual arrangements do you provide general practice? GMS  PMS

2. What is your contracted GMS/PMS commitment per week? (PLEASE TICK)

>26  19-26  13-18  Hours Job share

3. What percentage of a full partnership share do you receive? ___ %

(e.g. a full-time doctor might be on a 100% share, a part-timer 50% or 60%)

### YOUR CURRENT WORKLOAD

4. Please indicate the total number of sessions (half-days) (but NOT out of hours or Saturday morning) of all types of work (GMS and non-GMS) you do each week . Total number of **weekly sessions**

5. Please ***estimate*** how much of your time is taken up by the following types of work each week. Where activities are less frequent than weekly, please estimate the fraction in sessions per week, e.g. one 3.5 hour session each month would equate to 0.25 sessions per week.

| ***How many hours do you work each week in any of the spheres mentioned below. Please consider only normal DAYTIME work.*** | ***Number of sessions (if none write 0)*** |
| --- | --- |
| NHS CLINICAL GMS WORK |  |
| a) NHS General Medical Practice i.e. seeing patients in normal surgeries, house calls, clinics and associated day to day administration e.g. mail, letter writing, repeat prescriptions, etc. (EXCLUDING CATEGORIES BELOW) | . |
| NHS CLINICIAL NON-GMS WORK | |
| b) Locality or hospital specialty clinics | . |
| OTHER NHS/University NON-GMS WORK (tick all that apply) |  |
| c) Specific time set aside for  GP registrar training, (trainer, course organiser or associate advisor sessions)  Teaching medical students as tutor  Medical research  Practice administration/audit (e.g. as a lead partner NOT day to day admin)  Working for LHCC/PCT/ administration or as Appraiser  Protected learning time  Other  (please say what) | Total time spent on Other NHS/Univ  . |
| NON NHS WORK | |
| d) Sessional work outside practice (non NHS) e.g. industry or private general practice;Non GMS medicals, insurance reports etc. | . |
| **TOTAL** number of sessions | . |

6. How many hours per week (excluding out of hours on-call) do you  hours per week

spend working at home on General Practice related work?

7. In your view has the proportion of the time you have been available to devote to direct GMS patient care increased, decreased or stayed the same in the last **five** years? (please tick)

Increased  Decreased  Stayed the same  Not applicable - in practice less than 5 years

**PTO PTO PTO**

# THE FUTURE Please Tick

8) Do you plan to retire within the next 2 years? NO  go to question 9. YES  go to question 11

9) Do you plan to change your overall total number of daytime sessions per

week over the next 2 years (**NOT** including out of hours or Saturdays)?

a) NO (please tick) b)Don’t know  (please tick)

c) Increase by  sessions per week d) Reduce by  sessions per week

10. Do you see the time you spend on following areas in the next 2 years, in relation to your current workload staying the same, increasing or decreasing? Please tick the appropriate box.

|  | **Stay the same** | Increase | **Decrease** |
| --- | --- | --- | --- |
| **NHS CLINICAL GMS WORK**  a) General Medical Practice i.e. seeing patients in normal surgeries, house calls, clinics and associated administration e.g. letter writing, reading mail, repeat prescriptions etc. |  |  |  |
| NHS CLINICIAL NON-GMS WORK Specific sessions Locality or hospital specialty clinics |  |  |  |
| OTHER NHS/University NON-GMS WORK GP registrar training, (trainer, course organiser or associate advisor sessions), teaching medical students as tutor, medical research, practice administration/audit, working for LHCC/PCT/ administration or as appraiser, protected learning time Other |  |  |  |
| NON NHS WORK |  |  |  |

### RETIREMENT

11. What age are you now?  years 16. At what age do you hope to retire?  years

12. What proportion of your final salary do you hope to retire on?  % or don’t know (tick)

13. Will your *current* financial planning allow this (e.g. added years)?

**Please tick** Yes  No  Don’t know

### BACKGROUND INFORMATION

14. Are you male  or female  ? 20. Which NHS board do you practice in? __________________

15. What is your practice type? Inner city  Urban  Suburban  Rural  Isolated rural

16. What is the list size of your practice?

17. How many full-time equivalent principals work in your practice (e.g 3.75)? .

18.How many full-time equivalent associates (NOT GP Registrars) work in your practice? .

###### ADDITIONAL COMMENTS – please use an additional sheet for any other comments you wish to make
