# Supplementary material for: The feminization of the medical work force, implications for Scottish primary care: a survey of Scottish general practitioners
Source: BMC Health Serv Res. 2006 May 10;6:56. doi: 10.1186/1472-6963-6-56 (PMC1475570; doi:10.1186/1472-6963-6-56)
Supplement: Additional File 2 — Questionnaire for non-principals [file 1472-6963-6-56-S2.doc]

***Contribution to NHS and Non-NHS Work***

If you have just become a non principal in the past twelve months this section should reflect your work INTENTIONS for the next year. If you have been working as a non principal in the past twelve months please complete this part of the form reflecting on the work you have actually undertaken in the past year.

1. As a non principal, is all your NHS work undertaken in Scotland?

Yes  No

If no what proportion of your work is undertaken outside Scotland %

1. In addition to work in clinical general practice (in and out of hours), do you undertake any other NHS related work?

Yes  go to question 3

No  go to question 5

1. In relation to other **NHS** related work, what do you do?

Please fill all that apply

Hospital sessions

Educational (NES) sessions

Management – e.g. LHCC sessions

Research sessions

University sessions

Other (please specify)………………………….

1. Approximately, how many sessions do you contribute in these ways (i.e. **not** clinical general practice) per week?

<1  1  2  3  4  5  6  7  8  9  10

1. Do you undertake any **NON-NHS** related work?

Yes  go to question 6 No go to question 7

6. In relation to other **NON-NHS** related work, what do you do?

Please fill

DSS Medicals  Number of sessions per month

Insurance Medicals  Number of sessions per month

Private General Practice  Number of sessions per month

Occupational Medicine  Number of sessions per month

Other (Please state)…………………. Number of sessions per month

Total number of non-nhs sessions per month

7. In relation to clinical general practice, under what title(s) do you work? (you may fill more then one box):

Locum

Assistant

Associate

Fellow

Retainee

Other  please state ……………………………….

8. In relation to ***clinical general practice*** ***during working hours***, what was your average sessional commitment per week over the last year?: Please complete one box.

<1  1  2  3  4  5  6  7  8  9  10

9. In relation to clinical general practice did you contribute to ***out of hours*** work?

Please fill Please fill

Yes  go to 10 No to go to 11

10. What was your sessional commitment to out of hour’s work over the last 12 months?

Number of sessions

Evening (all days)

Overnight (all days)

Daytime (at weekend)

11. Do you intend to work in out of hours following the introduction of the new GMS contract?

Yes  go to 12

No  go to 13

13. Will your out of hours commitment:

Stay the same

Increase

Decrease

#### Career Intentions

14. In the next five years, what are you likely career intentions? (Complete all that apply)

Locuming  *Leave general practice

Salaried Assistant/Associate  *Leave NHS

Retainee  *Leave Medicine

Become a partner  *Leave UK

Other please say what  …………………………….

If you filled any of the options marked * please say why…………………………………………

15. If you are going to continue in your current roles, do you think your clinical commitment will:

### General Practice NHS (NON-GP) NON-NHS

Stay same  Stay same  Stay same

Increase  Increase  Increase

Decrease  Decrease  Decrease

## ***About You***

16. Are you ? Male  Female

17. How old are you  years

18. Where did you live for most of the year prior to entering medical school? (please fill one)

Scotland  Elsewhere UK

Eire  Other EAU

Outwith EAU  Please say where ………………….

18. Where did you go to medical school

Scotland  Elsewhere UK

Eire  Other EAU

Outwith EAU  Please say where ………………….

19. Which year did you qualify?

20. Which year did you complete vocational training (if applicable)?

Thank you for completing the questionnaire We really appreciate it. Please add any other comments here or on a separate page
